# Effects of Exercise in People with Severe Mental Illness and Recommendations for its Implementation as Add-on Therapy

**DOI:** 10.1192/j.eurpsy.2022.80

**Published:** 2022-09-01

**Authors:** I. Maurus, L. Röll, D. Keeser, A. Schmitt, A. Hasan, D. Hirjak, A. Meyer-Lindenberg, P. Falkai

**Affiliations:** 1 University Hospital LMU Munich, Clinic For Psychiatry And Psychotherapy, Munich, Germany; 2 University Hospital, LMU Munich, Department Of Radiology, Munich, Germany; 3 Universität Augsburg, BKH Augsburg, Psychiatry, Augsburg, Germany; 4 Zentralinstitut für Seelische Gesundheit, Dept. Of Psychiatry, Mannheim, Germany

**Keywords:** exercise, physical activity, schizophrenia, bipolar disorder, major depression

## Abstract

There are many reasons for people with (and without) severe mental illness to exercise regularly. In people with schizophrenia, major depression and bipolar disorder, it has already been shown that regular physical activity as an add-on therapy can improve quality of life and symptom severity. This is particularly important in domains that standard therapy is currently not able to treat sufficiently, such as cognitive deficits. Postulated underlying neurobiological effects include increased volume in hippocampal areas as demonstrated by data of a current clinical trial in people with schizophrenia.

Furthermore, regular exercise is essential to counteract the increased cardiovascular morbidity and mortality of people with severe mental illness. However, most people with severe mental illness do not achieve the recommended amount of physical activity and the potential of exercise as an add-on therapy is currently not even close to being fully realized. On the one hand, it is important that mental health staff also considers the physical condition of patients with mental illnesses and counsels them on their health behavior. On the other hand, there is a need for individually adapted training programs delivered by qualified exercise professionals that incorporate motivational and adherence strategies. Examples of barriers and facilitators for the implementation of exercise as an add-on therapy are discussed on the basis of current local projects.

**Disclosure:**

No significant relationships.

